# Characteristics of Fluorescence Emission Excited by Grating-Coupled Surface Plasmons

**DOI:** 10.1007/s11468-018-0757-8

**Published:** 2018-05-16

**Authors:** Andreas Nicol, Wolfgang Knoll

**Affiliations:** 1 Bayer AG, Engineering and Technology, Building E 41, 51368 Leverkusen, Germany; 2 AIT Austrian Institute of Technology, Konrad-Lorenz-Straße 24, 3430 Tulln an der Donau, Austria; 3 University of Natural Resources and Life Sciences, Vienna,(BOKU), Vienna, Austria

**Keywords:** Surface plasmon resonance, Surface plasmon field-enhanced fluorescence spectroscopy, Grating-coupled surface plasmon spectroscopy, Chromophore, Imaging

## Abstract

Dye molecules placed on metallic gratings can experience an enhanced electromagnetic field if illuminated under surface plasmon excitation conditions, a situation that can be employed for sensor applications. The fluorescence emission in this situation exhibits a characteristic emission polarization and geometry given by the fluorophore/grating interaction. We present experiments visualizing the full shape of the emission profiles and demonstrate how they can be manipulated by means of the grating constant. The excitation and emission processes taking place on the grating surface are characterized by polarization sensitive measurements.

## Introduction

Surface plasmon resonance (SPR) spectroscopy [1] has matured into a versatile method for the quantitative characterization of thin films and interfaces [2]. Recent reviews have demonstrated that there is still an enormous interest in further developing the fundamentals of this optical tool [3–5] and to describe new directions for various fields of applications [6–8].

The classical excitation of surface plasmons at a metal-dielectric interface by light using a glass or quartz prism was first described by Otto [9]. However, the alternative technique, introduced by Kretschmann [10], turned out to be a lot more versatile for widespread applications. The latter method uses a thin film, typically of a noble metal (Au or Ag) evaporated onto the coupling prism, with the surface plasmon mode being excited through the prism at the opposite metal/dielectric interface. Both approaches operate with photons in the total internal reflection geometry and thus meet the needs to match the energy and the momentum between photons and surface plasmons at resonant excitation.

Alternatively, the use of a surface corrugation of the metal surface, i.e., a grating structure, for the required momentum matching offers a number of advantages [1]: (i) by the appropriate choice of the grating constant *Λ* = 2*π*/*L*, with *L* being the grating periodicity, one can choose the angle of incidence for any wavelength at will [11]; (ii) similar considerations apply for the recording of scattered surface plasmons, resulting, e.g., from a Raman scattering process, that populate energetically lower-lying (Stokes shifted) plasmon modes. These modes then out-couple at a particular emission angle, again governed by the dispersion relation [12]; (iii) the equivalent holds for the use of surface plasmon modes for the excitation of the fluorescence of chromophores within the evanescent field of the mode [13]; (iv) for practical applications, certain optical material parameters, e.g., the thickness of the Au layer evaporated onto the prism in the Kretschmann configuration, need to be very well controlled. This can impose severe challenges for the fabrication process. Gratings are much more forgiving in that in most cases, the conformal deposition of a Au or Ag coating onto the substrate that carries the surface corrugation can easily vary in thickness by a factor of two; (v) the momentum matching by a lead Fourier component of a periodic surface corrugation, together with nano-structures that allow for the simultaneous excitation of localized surface plasmon modes (and combination excitations of propagating and localized modes), currently offers the largest field enhancements that can be applied for surface plasmon-based sensor platforms [14].

**Fig. 1:**
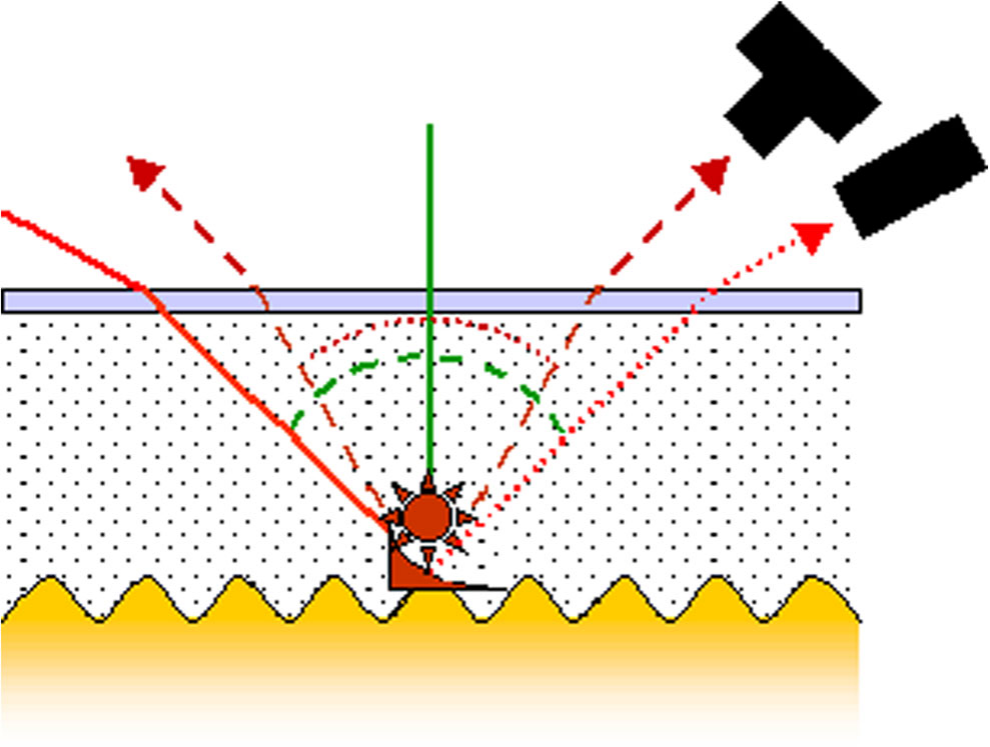
Excitation and detection geometry for grating-coupled SPR and SPFS. The sample is illuminated with a laser beam (solid line) through the flow cell volume. The intensity of the reflected beam (dotted line) is monitored in SPR. A dye immobilized on the grating emits fluorescence at distinct angles (dashed lines). This intensity is the relevant signal in SPFS

Already a long time ago, a first report on the fluorescence emission of dyes placed on a periodically structured metal surface has been published [15]. Only a surprisingly small number of articles have been published since then on this subject. These cover calculations of the plasmonic fields and enhancement optimization [16], various excitation and emission geometries [17], studies of fluorescence lifetime as a function of the dye/metal separation [18], and applications in biosensing [19]. We examine here the emission geometry of the excited fluorescence and the influence of the polarization (cf. Fig. 1). Previous papers have already mentioned the strongly directional character of the fluorescence emission [20]. This study maps the emission characteristic in the azimuthal as well as in the polar range. The grating/dye interaction in both the excitation and the emission process is examined by means of polarization-dependent measurements. A labeled DNA immobilization assay was employed as a model biosensor surface functionalization (Fig. 4) [21].

## Experimental

### Grating Manufacture

**Fig. 2:**
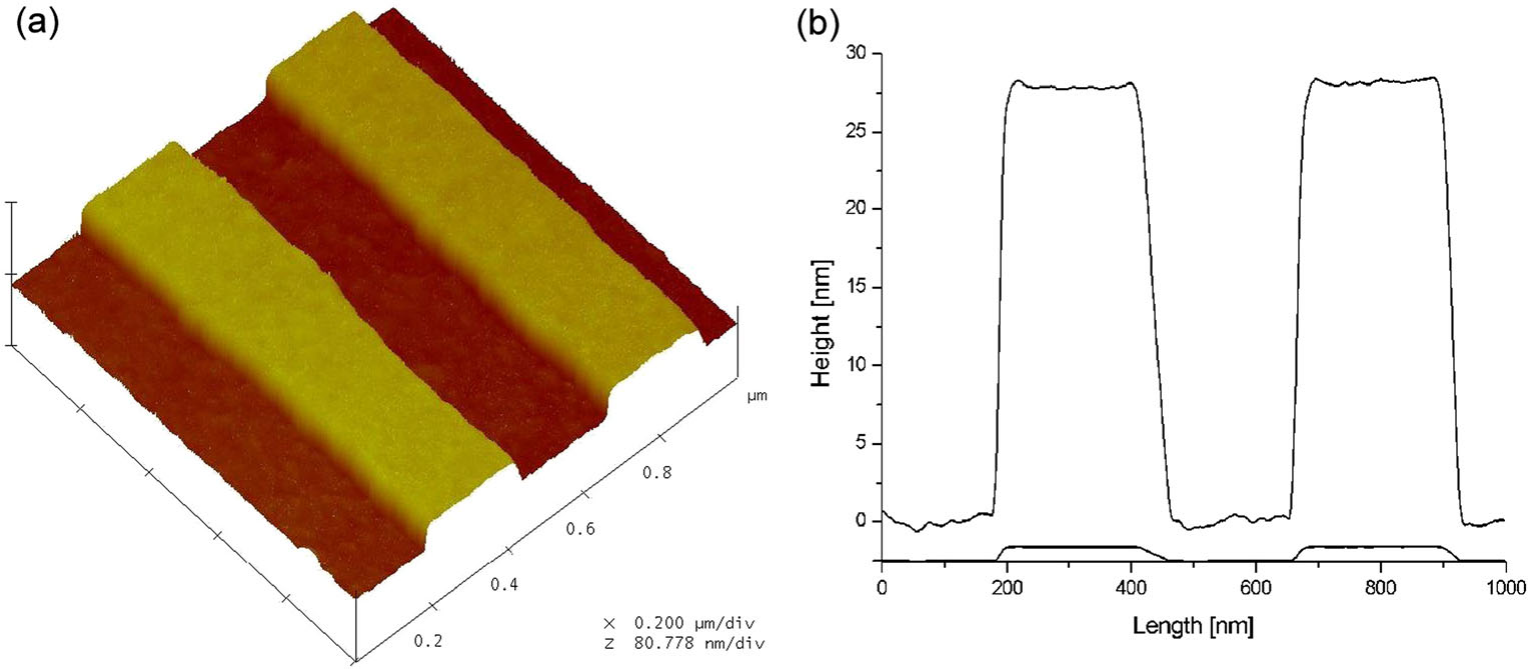
Topography of an un-metalized grating taken by AFM. **a** The three-dimensional view shows a 1 μm × 1 μm area from the center of the glass sample B104. **b** The section plot is based on averaging over the data of the full area. It yields a trapezoidal type of profile (*Λ* = 478.5 nm) with an amplitude of *H* = 13.9 nm. The smaller curve of the two shows the profile for equally scaled axes and illustrates the shallow character of the corrugation

Grating structures were manufactured holographically [22]. A photoresist film spin cast unto a glass substrate was exposed to an interference pattern of a laser beam. After development, the pattern was transferred into the substrate material by reactive ion beam etching [23, 24]. The samples were evaporation-coated with 2 nm of chromium and 150 nm of gold. Substrates in this study were designed with a mean amplitude of *H* = 15.2 nm and a grating constant of *Λ* = 474.7 nm, verified by scanning probe topography images (cf. Fig. 2) and measurements of grating diffraction in the Littrow mount, respectively [25].

### Surface Functionalization

**Fig. 3:**
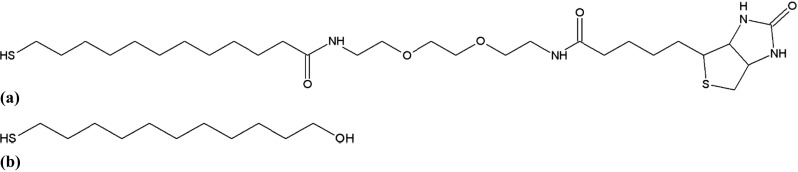
**a** Biotinylated thiol: 12-mercapto(8-biotinamide-3,6-dioxaoctyl)dodecanamide. **b** Spacer thiol: 11-mercapto-1-undecanol

The purpose of the surface functionalization by a self-assembled monolayer (SAM) is twofold: to provide (i) sensor specificity [26] and (ii) optimal spacing between fluorophores and metal layer [20]. Samples were functionalized in order to exhibit oligonucleotide recognition sites according to the assay described in [21]. In short, a monolayer of two different thiols was self-assembled on the corrugated gold surface first. One thiol species was end-functionalized with a biotin group (Fig. 3a) and used for subsequent binding steps. The other OH-terminated thiol (Fig. 3b) served as a lateral spacer and was selected so as to minimize non-specific adsorption.

**Fig. 4:**
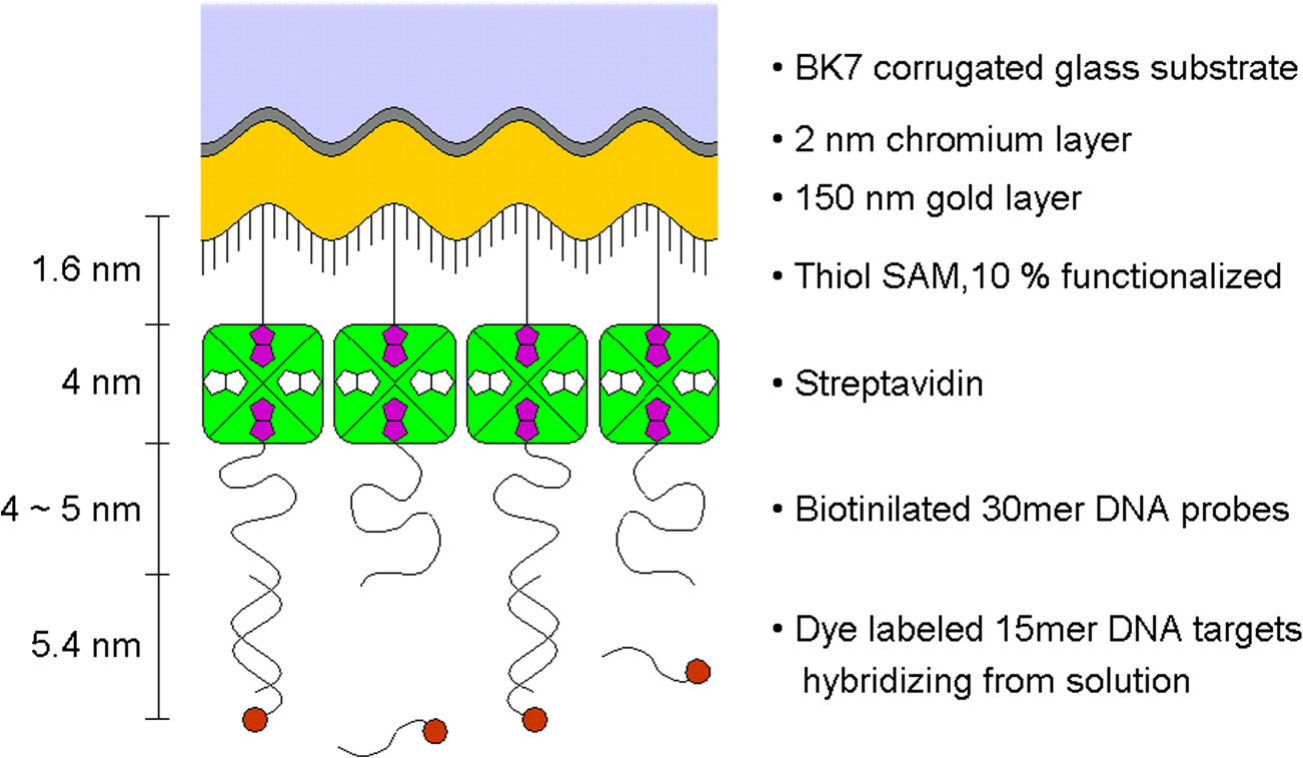
Biomodification of the sensor surface. The figure sketches the individual layers of the functionalization architecture. All features are schematic and not to scale. The streptavidin/biotin binding geometry and stoichiometry are also meant to be schematic. The gratings were of very small aspect ratio (cf. Fig. 2) and the subsequent layers are following the underlying undulations

Next streptavidin was bound to the biotinylated thiol SAM. Its remaining binding pockets were used to immobilize a biotin-terminated 30-mer DNA probe. The last 15 nucleotides of the strand constituted the recognition sequence. Fluorescently labeled DNA target strands (one molecule of Cy5 at the 5′ end, *λ*_abs_ = 646 nm, *λ*_em_ = 664 nm) with a complementary sequence were immobilized from 500 nM solutions in order to saturate the surface quickly. The complete surface architecture is illustrated schematically in Fig. 4.

### Experimental Setup

**Fig. 5:**
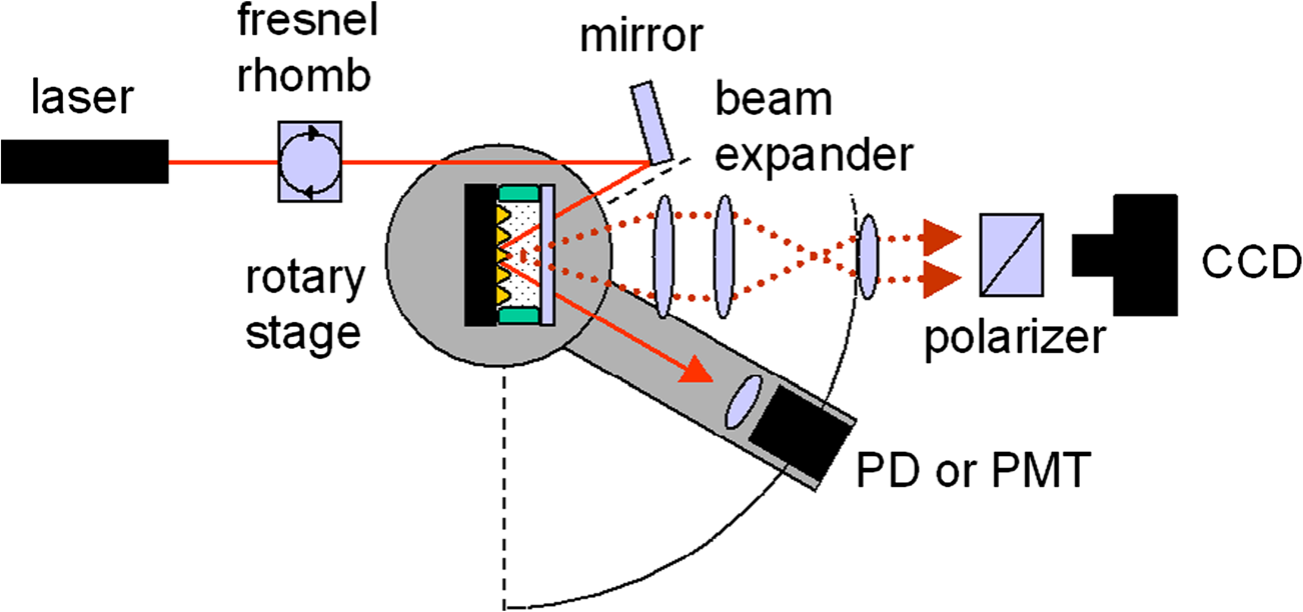
The experimental setup. The sample was mounted in a flow cell on a rotary stage to allow for scanning of the angle of incidence. It was illuminated by a chopped HeNe laser beam with tunable polarization by means of a Fresnel rhomb. The intensity of the emitted fluorescence light was recorded with a cooled CCD camera covering the full solid angle of the directed emission. The second arm of the rotary stage could move a photodiode for measuring the reflected beam intensity or alternatively a PMT for the pixel-to-angle calibration of the CCD chip

Figure 5 shows the setup schematically. A HeNe laser (*λ* = 632.8 nm, 5 mW, PL-750P polarized helium-neon laser, Polytec) was used as the excitation source. The plane of polarization could be set by a Fresnel rhomb (Fresnel rhomb *λ*/2, Bernhard Halle Nachfl.), while keeping the output power constant. Besides the obvious choice of TM polarization that is required for surface plasmon excitation, TE polarization could also be used to distinguish contributions from direct illumination from those generated by the evanescent field. Next, the beam was reflected onto the sample via a mirror rather than being routed directly. This reflection beam steering was chosen to decouple the geometric constraints of the excitation beam and the recording CCD camera, thus minimizing the blind angle.

Gratings were mounted inside a home built flow cell connected to a peristaltic pump on one of the arms of two rotary stages (Model 414A00 two-circle goniometer with M-BF-810-12 stepper motors, HUBER Diffraktionstechnik). By rotating the sample, the angle of incidence *θ* could be changed. The second arm carried a photodiode (PD). The PD (BPW 34 B silicon photodiode, Siemens) recorded the photocurrent generated by the light reflected from the grating for reflectivity measurements.

The fluorescence detection capability was provided by a cooled CCD camera (CoolSNAP_HQ_ Monochrome, Photometrics) positioned with its optical axis oriented parallel to the surface normal of the grating and above its center. The fluorescence emission was turned into a parallel pencil of rays first and then shrunk in solid angle by a Kepler beam expander to fit the sensitive area of the CCD using a paraxial series of three lenses between sample and camera. The light had to pass through two interference filters (670FS10-25 interference filter, transmission maximum 67% at 673 nm, LOT Oriel) matched to the fluorescence wavelength and, for certain experiments, also a polarizer before it reached the detector. Images obtained with the CCD camera are rather two-dimensional angular spectra (a position on the CCD image relates to the angle under which the ray has left the grating) than images in the strict sense (a position in the object plane corresponds to a position in the image plane). Additionally, a photomultiplier tube (PMT) was used with an identical interference filter. With the CCD camera removed, the PMT (H6240-01 photon-counting unit, Hamamatsu) could be mounted on the second goniometer arm instead of the PD. It was used to measure the fluorescence intensity as a function of azimuthal angle in order to calibrate the horizontal axis of the CCD chip.

### Experimental Methods

The surface functionalization of the grating substrates was built sequentially. Immobilization of each layer except the first was monitored by kinetic SPR measurements. The minimum position of the reflectivity curve taken after the addition of the probe sequence gives the angle of incidence for resonant excitation. The sample is then kept fixed at this angle in the following set of experiments. Next, the fluorescently labeled target sequence was injected and cycled for 5 min to guarantee a saturated sensor surface. After this, the flow cell was rinsed with PBS buffer in order to remove free fluorophores. The PD was exchanged against the PMT (with the CCD camera removed in order to free up the required space) to scan the (azimuthal) emission angle relative to the grating normal. Snap shots of the solid angle of emission were taken after having mounted the CCD camera assembly with its optical axis perpendicular to the grating surface.

## Results and Discussion

### Emission Profile Versus Grating Constant

**Fig. 6:**
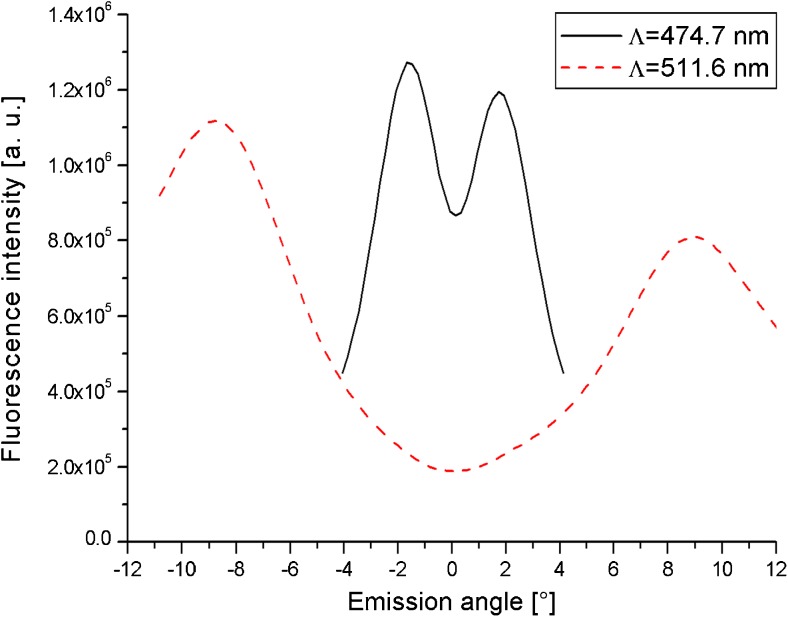
Tuning the emission angle by changing the grating constant. The first sample (solid line) has a grating constant of *Λ* = 474.7 nm. It was resonantly excited at *θ* = 8.06°, yielding a maximum in the fluorescence emission pattern at *Δθ* = ± 1.75° relative to the grating normal. The other grating (*Λ* = 511.6 nm) is resonantly excited at *θ* = 15° and shows a larger angular separation between the two fluorescence emission cones with peaks at about *Δθ* = ± 9°

Angular scans of the fluorescence intensity were recorded with the PMT before taking measurements with the CCD camera. Figure 6 shows two such curves for samples with different grating constants, *Λ* = 474.7 nm and *Λ* = 511.6 nm, respectively, demonstrating how the device geometry can be engineered by tuning the grating constant. In both cases, the illumination was TM-polarized and the full emission was recorded, irrespective of polarization. The curves indicate the strong directional character of the fluorescence emission. The reason for this lies in the fluorophore/grating interaction during both, the excitation and the emission process. In the excitation step, the incident beam couples to surface plasmon modes and energy is transferred from the evanescent field of a surface plasmon to a fluorophore. The same mechanisms can take place with time being reversed: an excited fluorophore can transfer its energy into a metal film to launch a red-shifted surface plasmon, which then decays via emission of a far field photon at the fluorescence wavelength. Because the re-radiation is now bound to the coupling condition of matching plasmonic and photonic momenta, this back-coupled fluorescence emission shows a distinct angle dependence. Lately, back coupling has been studied in great detail in the context of prism coupling geometries [20].

Changing the grating constant affects the peak emission angle, because a different grating vector is introduced to the momentum-matching condition in reciprocal space. The curve obtained from a sample with a grating constant of *Λ* = 474.4 nm features lobes that are located closer to each other than for the sample with a higher grating constant. At the same time, the width of the peaks is much smaller. By picking the right grating constant, the emission angles can be engineered in a way that is convenient for a given device geometry. Unfortunately, the fluorescence intensities generated by both samples could not be compared in a quantitative way. Samples with a grating constant of *Λ* = 474.7 nm only became available much later in this work. At that time, certain properties of the experimental setup, like beam collimation and thus laser power, had been changed.

The two curves in Fig. 6 are characterized by an apparent asymmetry of the two emission peaks which is an artifact caused by bleaching of immobilized dye molecules and not a consequence of a grating corrugation asymmetry or another anisotropy effect. Typically, curves like those shown required a measurement time (and hence exposure to the laser beam) of 5 to 10 min, depending on the desired angular coverage. Reversal of the scan direction reversed the asymmetry.

### Shape of the Fluorescence Emission Peaks

**Fig. 7:**
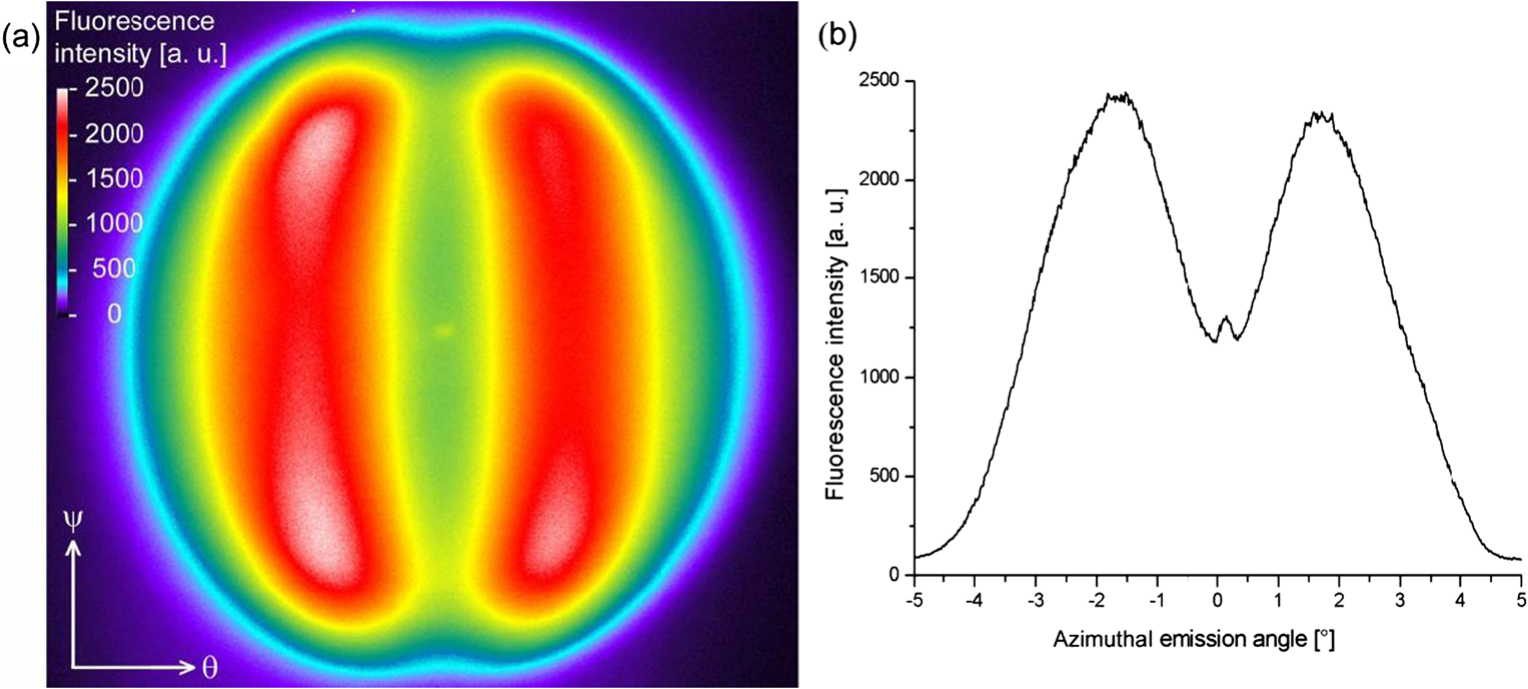
Imaging the fluorescence profiles. **a** Imaging the solid angle of the fluorescence emission shows the two high-intensity lobes emitted from a sample under resonant excitation at *θ* = 8.45°. **b** A horizontal (azimuthal plane) line scan through the center of the image. The ordinate scale in **b** can be directly mapped onto the fluorescence image given in **a**. The data is almost perfectly symmetric around the center of the image. Close to the center, a little spike close to the origin appears which is thought to be caused by a multiple reflection of the laser beam

**Fig. 8:**
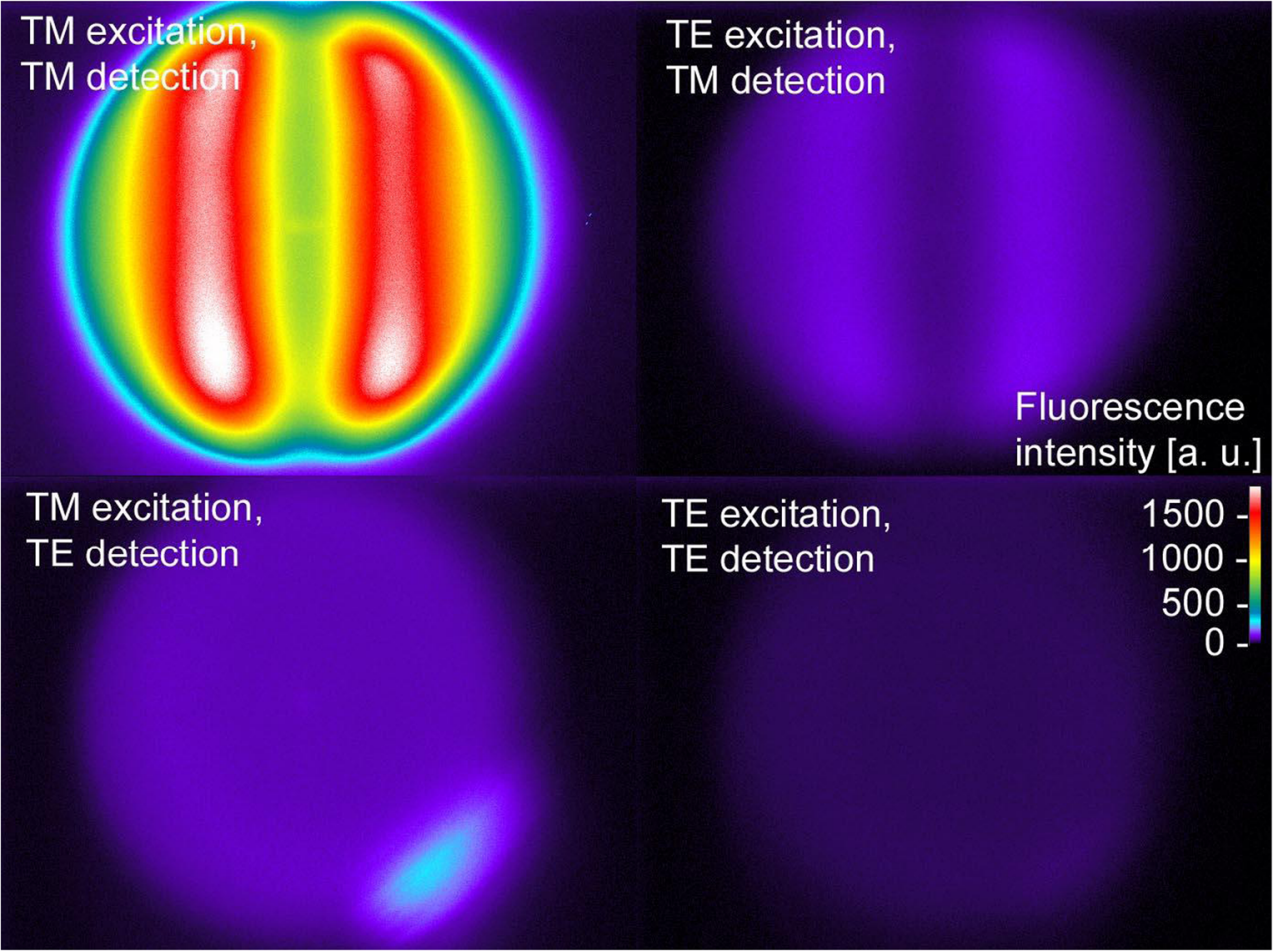
Solid angle images of polarization sensitive detection. The strong directional character of the TM-polarized fluorescence emission is clearly visible under TM light excitation. The two emission lobes are also visible if the illumination is TE-polarized, but at much lower intensities. The intensity spectra recorded for the two combinations of crossed excitation and detection polarization (TM/TE and TE/TE) are almost isotropic. The blurred spot in the lower right corner of the TM/TE image was caused by a dust particle

**Fig. 9:**
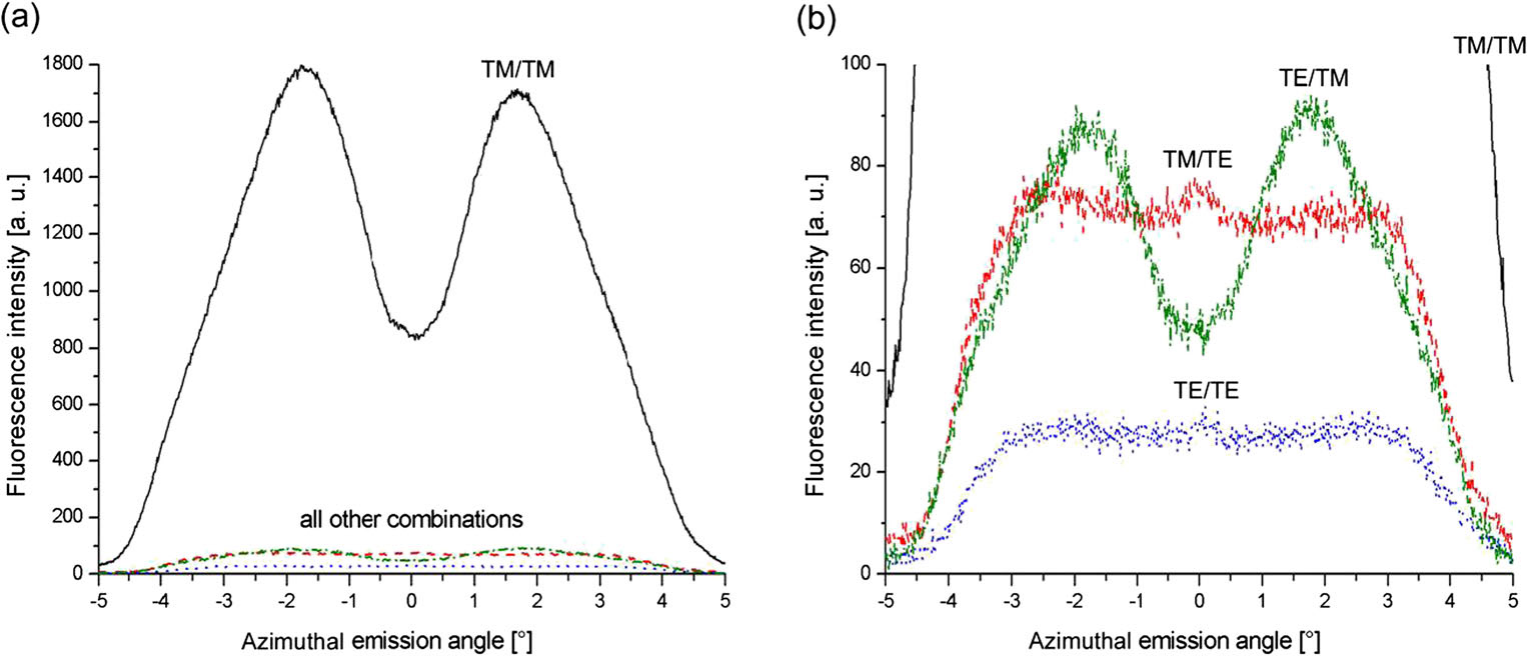
Line scans of polarization-sensitive detection. All four curves represent horizontal line scans through the center of the images shown in Fig. 8. The ordinate scales in all scans presented in **a** and **b** can be directly mapped onto the fluorescence image given in Fig. 8 **a** All four combinations of excitation and emission are given in the graph, with TM/TM exhibiting the strongest intensity. **b** This graph shows the same data in more detail, focusing on the three weaker combinations. Independent from the excitation polarization, the emission of TM-polarized fluorescence is always directional and hence predominantly grating-coupled. In contrast to that, TE-polarized emission is almost isotropic within the field of view of the system

Artifacts due to anisotropic bleaching can be eliminated by recording the fluorescence emission of a sample with a single snapshot (15 s exposure time) of a CCD camera, the trade-off being its lower signal-to-noise ratio compared to the much more sensitive PMT technology. This solid angle imaging (SAI) method can map the full shape of the fluorescence emission lobes as shown in Fig. 7 for a different sample. The two lobes are visible as the two kidney-shaped bright regions. They are well separated by the polar plane at *θ* = 0° where the fluorescence intensity assumes a local minimum. The circular boundary of the image is caused by the aperture limits of the expansion optics resulting in a cut-off at approximately *Δθ* = ± 3°. With respect to this type of measurement, the plots of the fluorescence intensity versus azimuthal angle like in Fig. 6 are horizontal line scans through the center of the solid angle image (compare Fig. 7b). Both lobes are of almost equal intensity. The minute difference between the two peaks visible in the figure is attributed to small imperfections in the optical alignment. Other samples and measurements can exhibit peaks of equal height or the inverted height relationship (see below). The spike close to the origin is attributed to a multiple reflection of the laser from the flow cell that finds its way through both interference filters into the detector. It only appeared if a flow cell was mounted and was already observable before injection of the dye, independent of the illumination polarization. Because this spike is not related to fluorescence emission, it can be ignored in Figs. 7, 8, and 9.

### Polarization Sensitive Measurements

The fluorescence measurements in the previous experiments were performed in a polarization insensitive way, by recording the total intensity without discrimination between TM-polarized and TE-polarized fluorescence light. In the following experiments, a polarizer was mounted right in front of the CCD camera. The polarization of the illuminating beam was controlled by the orientation of the Fresnel rhomb. Therefore, four combinations of polarizations are possible: excitation by TM or TE and detection by TM or TE. These combinations are abbreviated in the form a/b from here on, where a is the polarization of the incident light and b is the polarization state interrogated by the CCD. Solid angle images from measurements performed under these four polarization combinations are presented in Fig. 8.

Azimuthal plane line scans through the center of these images are compiled in Fig. 9. The TM/TM combination results in the highest intensity for the full imaged range of angles and exhibits a strong directional character. Excitation and emission both utilize the grating and are therefore enhanced. The configuration TE/TE results in the lowest signal and is virtually isotropic across the covered angular range, because of the lack of interaction with the grating. Similarly, intensity profiles in the TM/TE case are almost unstructured. This combination of polarizations describes the emission from a small fraction of fluorophores that are excited by the evanescent field, but their orientation changes before fluorescence emission can take place and they radiate freely without coupling to the grating. By the same token, dye molecules excited by TE light can depolarize and emit in the perpendicular polarization state. Because of the enhanced emission via the grating, this TE/TM combination is even stronger in intensity than TE/TE.

## Conclusion and Outlook

The enhanced electromagnetic field in grating-coupled surface plasmon excitation has been used in this work to excite fluorophores. The fluorescence emitted from a dye-coated grating was found to be strongly directional. The emission geometry was recorded by solid angle imaging. It appears as a double hump symmetric to the surface normal of the sample. We demonstrate how the shape of the emission lobes can be manipulated by varying the grating constant. It is further shown by means of polarization-dependent excitation and detection that this shape is the result of the excited fluorophores, relaxing via intermediate red-shifted surface plasmons, which decay radiatively via the grating. This process of double-grating interaction is favored over other techniques employing, e.g., prism coupling, and achieves double enhancement.

We have examined fundamental properties of grating-coupled SPFS targeted towards its application for biosensing. In future publications, we will demonstrate the in situ fluorescence detection of analyte immobilization, quantify the impact of emission from fluorophores in the bulk on the limit of detection, and describe strategies to compensate this bulk signal contribution.
